# Achieving objective response in treatment of non-resectable neuroendocrine tumors does not predict longer time to progression compared to achieving stable disease

**DOI:** 10.1186/s12885-020-06963-6

**Published:** 2020-05-24

**Authors:** Espen Thiis-Evensen, Amalie Christine Poole, Hong-Thien Thi Nguyen, Jon Sponheim

**Affiliations:** 1 Center for neuroendocrine tumors, Department of Gastroenterology, Oslo University Hospital, Rikshospitalet, Sognsvannsveien 20, 0424 Oslo, Norway; 2 Faculty of Medicine, University of Oslo, Oslo, Norway

**Keywords:** Neuroendocrine tumor, Treatment, Radiology, Peptide receptor radionuclide therapy, Chemoteraphy

## Abstract

**Background:**

There are several treatment modalities for unresectable neuroendocrine tumors. Traditionally, the aim of these treatments has been to reduce the tumor load; referred to as objective response (OR). Less emphasis has been put on inducing the tumors to stop growing without a reduction in total tumor load; termed as stable disease (SD). We wanted to investigate whether achieving OR compared to obtaining SD predicted a longer time to progression (TTP) in patients with neuroendocrine tumors (WHO Grade 1 and 2) treated with peptide receptor radionuclide therapy, chemotherapy or molecular targeted therapy.

**Methods:**

Patients treated with either peptide receptor radionuclide therapy (PRRT) with ^177^Lutetium-DOTA-octreotate, the chemotherapy combination streptozotocin/5-fluorouracil or everolimus were retrospectively assessed to evaluate the effect of the treatments on disease progression. We analyzed the TTP for patients for each treatment modality and compared the TTP between those who achieved OR and those who achieved SD.

**Results:**

Altogether 56 patients treated with PRRT, 32 treated with streptozotocin/5-fluorouracil and 52 treated with everolimus were included in the analyses. The median TTP for those treated with PRRT and achieving OR was 31 months, the TTP for those achieving SD was 43 months (*p* = 0,2). For patients treated with streptozotocin/5-fluorouracil the results were: OR: 18 months, SD: 23 months (*p* = 0,9) and for those treated with everolimus; OR: 9 months, SD: 20 months (*p* = 0,5), respectively. We found no differences between patients achieving OR compared to SD regarding age, sex, stage, primary tumor location, Ki-67% or ongoing treatment with somatostatin analogues.

**Conclusions:**

We found no treatment benefit with regard to TTP for our patients that experienced OR compared to those who achieved SD.

## Background

Neuroendocrine tumors (NET) are a heterogeneous group of tumors arising from neuroendocrine cells. Their incidence is increasing worldwide. In Norway the registered increase has been from 5.3 per 100.000 in 1993–2001 to 7.0 in 2006–2010 [1]. Most NETs, about 70%, arise from the gastro-entero-pancreatic system [1, 2]. Surgery is as of today the only treatment modality that can cure the patient. More than 50% of the patients, however, presents with unresectable disseminated disease, often as an incidental finding. There are several treatment modalities that have been shown to reduce the tumor load or stop tumor growth. The most commonly used are somatostatin analogues, molecular targeted therapy (everolimus, sunitinib), peptide receptor radionuclide therapy (PRRT), and chemotherapy [3–8]. Traditionally, studies reporting the effect of these treatments have put most emphasis on the tumor load reducing effect (objective response; OR). Less emphasis, until recently, has been put on the treatments ability to stop tumors growth without necessarily reducing the total tumor volume (disease stabilization, stable disease; SD). In our clinical practice, we have had the impression that NET patients who have an objective response often seem to have a shorter time to progression than those who achieve disease stabilization. We wanted to investigate whether there was a difference in the time to progression (TTP) for patients with non-curable neuroendocrine tumors that experience an OR compared to those with SD.

## Methods

### Patients

All patients were treated at our center, a European Neuroendocrine Tumor Society (ENETS) accredited Center of Excellence in the treatment and care for patients with NETs, with a catchment area of 2.8 million people. We identified all patients treated with PRRT, the chemotherapy combination streptozocin/5-fluorouracil (stz/5-FU) or everolimus and the radiological response was retrospectively evaluated.

### Radiological assessment

The radiological response evaluation was usually performed 6 months after the last cycle of PPRT-treatment and thereafter every 6 months. For patients treated with stz/5-FU and everolimus, response evaluation was performed every 3 months. The evaluation was performed with contrast-enhanced CT-scans with arterial and portal venous phase or with contrast-enhanced MRI. With our routine evaluation of treatment effect, termed the “conventional method”, progressive disease is defined as detection of new lesions or any unequivocal increase in the size of known tumors, based on measurements of the individual lesions, when comparing examinations comparable in quality and performed with the same modality and same protocols for contrast enhancement. With this method changes in diameter of a lesion of 1–2 mm were not considered as significant due to minute differences in contrast enhancement between examinations and to small operator differences in performing the measurement of the lesions. Treatment response was defined as objective response (any unequivocal shrinkage of tumors, OR) or stable disease (no changes in number or size of tumors, SD). For patients treated with PRRT radiological response evaluation was in addition to the “conventional method” also performed according to the RECIST 1.1 criteria [9]. These RECIST assessments were performed by one single experienced senior oncology-radiologist.

### Stz/5-FU

This chemotherapy combination was mainly given to patients with pancreatic NETs as first-line treatment. The chemotherapy was administrated as a 5-days induction course followed with one-day cycles every 3 weeks. Seventy-two patients were evaluated. They received their treatment between April 2007 and May 2017. The best treatment effect based on radiological assessment was OR in 27 (38%), SD in 16 (22%) and progressive disease (PD) in 29 (40%) patients. Median progression-free survival was 11 months.

### PRRT

At our institution PRRT is usually given as second- or third-line treatment. Altogether 79 patients were treated with a median of 4 cycles with ^177^Lutetium-DOTA-octreotate [9]. They received their PRRT treatment in the period of January 2006 to March 2014. The best treatment effect based on radiological assessment using the “conventional method” was OR in 42 (53%), SD in 17 (22%) and progressive disease (PD) in 20 (25%) patients. Median progression free survival was 28 months. If the RECIST 1.1. was applied, the best effect was OR in 13 (17%), SD in 54 (68%) and progressive disease (PD) in 12 (15%) patients. Based on the RECIST 1.1., the median progression free survival, was 33 months.

### Everolimus

Everolimus is used as a second- to fifth-line of treatment and a total of 98 patients who received treatment between December 2008 and September 2017 were evaluated. The best treatment effect based on radiological assessment was OR in 15 (15%), SD in 45 (46%) and progressive disease (PD) in 14 (14%) patients. Median progression-free survival was 8.2 months.

### Inclusion criteria

The inclusion criteria were tissue sample verified neuroendocrine tumor with Ki-67% assessment, WHO grade 1 or 2 (Ki 67 20% or below), metastatic or non-resectable disease, lesions measurable on radiological evaluation, and at least one radiological evaluation after initiating therapy (stz/5-FU, everolimus) or after completed all planned cycles of PRRT. If the same patient had several tissue samples taken, the one with the highest Ki-67% was used to define WHO grade. Only patients with OR or SD as best radiological treatment response (based on the “conventional method”) were included.

### Statistics

Time to progression (TTP) was calculated for all study groups. For the PRRT cohort TTP was calculated both for the best treatment response based on the “conventional method” and for the best treatment response based on RECIST1.1. Log-rank test was used to compare survival curves, Mann-Whitney U-test was used to compare continuous variables, Chi-Square (or Fisher’s Exact test when appropriate) was used for testing categorical variables. A *p*-value below 0.05 was considered statistically significant. Inter-quartile range, the range from the 25th to the 75th centile, was used to present the range in Ki-67% estimates. As this study was exploratory no statistical power analyses were performed. The statistical analyses were performed using SPSS 23.0 software (SPSS Inc., Chicago, Ill.).

## Results

**Fig. 1:**
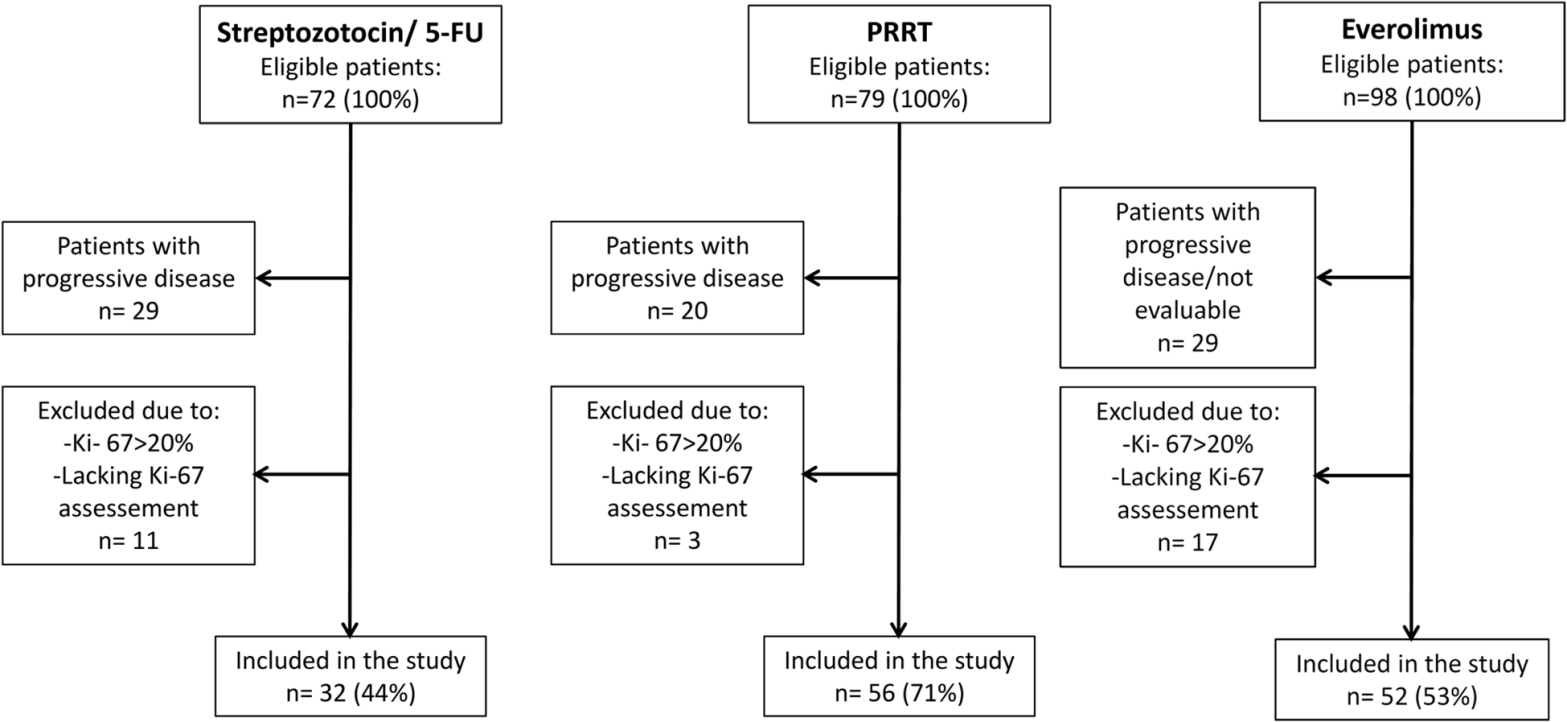
Flowchart showing the selection of patients from the original studies

In the stz/5-FU group 32 patients, in the PRRT treated group 56 patients and in the everolimus group 52 patients fulfilled the inclusion criteria (Fig. 1). The distribution of gender, age, primary tumor location, stage, previous treatments and ongoing treatment with somatostatin analogues are given in Table 1. Pancreas and the small intestine were the most common primary sites comprising altogether 74% of the study cohort. Pancreas as the primary tumor location dominated in the group treated with stz/5-FU, comprising 67% of the patients. Almost all patients had distant disease. Stz/5-FU was mostly used as first-line treatment, PRRT third-line and everolimus as fourth-line treatments. Only 1 patient in the stz/5-FU group had previously been treated with PRRT or everolimus whereas 43 (83%) in the everolimus group had previously been treated with stz/5-FU or everolimus (Table 1).

**Table 1:** Patient characteristics

	Stz/5-FU *n* = 32	PRRT *n* = 56	Everolimus *n* = 52
**Age,** years Median (range)	65 (28–83)	63 (29–79)	66 (41–81)
**Sex**			
Female	20 (63)	26 (46)	31 (60)
**Primary focus (%)**			
Pancreas	22 (67)	18 (32)	17 (33)
Small intestine	1 (3)	26 (46)	19 (37)
Lung	3 (9)	1 (2)	9(17)
Rectum	1(3)	3 (5)	
Kidney		1(2)	
Duodenum	1(3)	1(2)	
Pheochromocytoma		1(2)	
Gastric	1(3)		
Thymus			1(2)
Unknown	3 (9)	5 (9)	6 (12)
Stage			
Regional	2 (6)	1 (2)	4 (8)
Distant	30 (94)	55 (98)	48 (92)
Previous treatment with PRRT	1 (3)	–	25 (48)
Previous treatment with stz/5FU	–	12 (21)	18 (35)
Previous treatment with everolimus	1 (3)	1 (2)	–
Previous treatment with stz/5FU and PRRT	–	–	20 (38)
**Number of previous treatments**^**a**^ Median (mean)	0 (0,6)	1,9 (2,0)	3 (2,6)
**Follow-up time** Months (min-max)	47 (5–113)	48 (10–98)	14 (4–77)

Patient demographics, site of primary and previous treatments with PRRT, Stz/5FU and everolimus, (percent) and follow-up time (from last CT before initiation of therapy to death or end of study)

^a^ Includes all types of tumor targeted treatments, including surgery

**Fig. 2:**
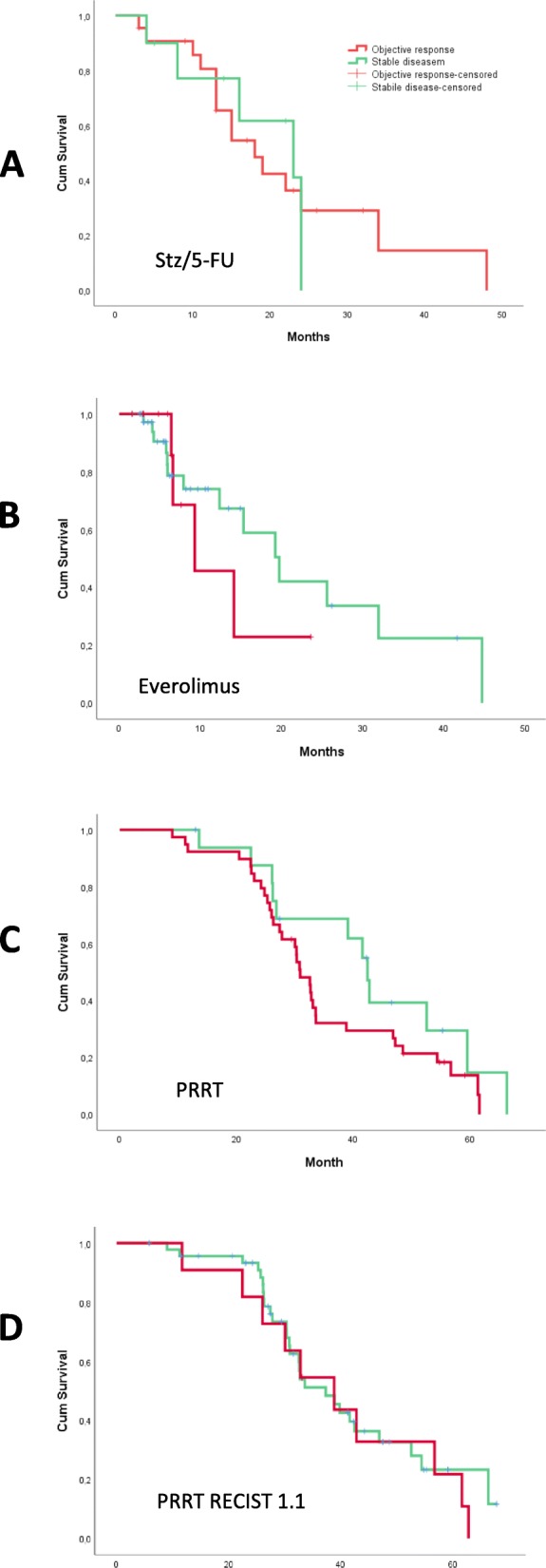
Time to progression. Time to progression in months for patients achieving stable disease (SD) and objective response (OR) treated with (**a**): streptozotocin/5-FU, (**b**): everolimus and (**c**) and (**d**): PRRT. Radiological response evaluation is done with the “conventional method” in (**a**), (**b**) and (**c**), where any unequivocal change in the the size of known tumors are considered significant. In (**d**) the response evaluation was done according to the RECIST 1.1 criteria

In the group treated with stz/5-FU the median TTP for those who achieved objective response was 18 months (95% confidence interval (CI) 12–24), and for those who obtained stable disease 23 months (95% CI: 9–36), *p* = 0.8 (Fig. 2). The same figures for those who achieved objective response compared to stable disease in the PRRT group were 43 months (95% CI: 41–44) compared to 31 (95% CI:28–34) *p* = 0,2, and for the everolimus group 9 months (95% CI: 2–17) compared to 20 months (95%CI: 13–26) *p* = 0,5, respectively. If the RECIST criteria were applied for response evaluation in the PRRT treated group instead of the “conventional method”, the median TTP for those who achieved OR compared to those with SD was 39 months (95%CI: 25–52) and 37 months (95%CI: 29–45), *p* = 0,6, (Fig. 2). When we compared the factors age, sex, Ki-67% and stage between those with OR and those with SD, we found no statistically significant differences or trends (Table 2). For those treated with stz/5-FU and PRRT, a larger proportion of women than men obtained OR, but for everolimus it was vice versa. However, these differences were not statistically significant.

**Table 2:** Treatment effects

	Streptozocin/5-FU			PRRT			Everolimus		
	OR *n* = 22	SD *n* = 10	*p*-value	OR *n* = 39	SD *n* = 17	*p*-value	OR *n* = 12	SD *n* = 40	*p*-value
**Age** years, median mean)	63 (63)	65 (65)	1.0	62 (62)	65	0,2	65 (65)	67 (66)	0,9
**Sex,** female (%)	10 (83)	2 (17)	0,2	20 (77)	6 (23)	0,2	2 (17)	19 (48)	0,06
**Ongoing SSA treatment** (%)	3 (14)	0	0,5	21 (54)	10 (59)	0,7	7 (58)	25 (63)	0,8
**Prior treatments** Median (mean)	0 (0,6)	0 (0,6)	1,0	2 (1,8)	2 (2,2)	0,1	2,5 (2,4)	3 (2,7)	0,7
**Ki67%** Median (IQR)									
Total group	10 (10–13)	10 (3–13)	0,2	7 (3,5–10)	6 (1–10)	0,4	9 (3,5-11,5)	9 (5–12)	0,7
Pancreas	10 (8–13)	10 (10–14)	0,7	7 (4–12,5)	10 (5,5–10)	1,0	8 (5–13)	10 (8,5-12,5)	0,5
Small intestinal	–	–	–	6 (2,5-11,5)	3 (1–8,5)	0,4	6 (1,5–10)	7 (3–11,5)	0,4
**Stage** No. patients (%)									
Regional	1 (5)	1 (10)	0,5	1 (3)	0	0,5	1 (8)	3 (8)	0,9
Distant	21 (95)	9 (90)		38 (97)	17 (100)		11 (92)	37 (92)	

Age, sex, ongoing somatostatin analogue-treatment, prior treatments, Ki 67% and stage for from patients divided into treatment modality and best treatment response; objective response or stable. For Ki 67% data is given for the total patient group and further subdivided into the most common primaries; pancreas and small intestine. *SSA* somatostatin analogue, *IQR* interquartile range

## Discussion

In this study with patients with neuroendocrine tumors grade I-II treated with several classes of tumor targeted treatments, we did not find any statistically differences in TTP between those who achieved OR and those who achieved SD. There was, however, a trend towards shorter TTP for those who achieved OR. We have not been able to find any other study that systematically compare the TTP in response groups (OR or SD) in tumor targeted therapies. We found no differences in patient- or tumor characteristics that separated the two response groups. Age, Ki 67%, site of primary tumor location and stage were comparable within the two response groups. This may indicate that there could be other biological factors than Ki67% and the known behavior of the different primaries that could influence both the response and the duration of the response to tumor targeted treatment modalities.

The strength of this study was that all patients were well characterized, treated in a single center and none of the patients were lost to follow up. The study has, however, several limitations. The numbers of patients in each group were few and the power to detect differences between the response groups low. Some patients were included in more than one group, i.e. 83% in the everolimus group had previously been treated with stz/5-FU or PRRT, and 38% had received both treatments. This might lead to a selection bias reproducing the same pattern with those with SD tending to have longer TTP for the different treatment modalities studied. Still, this did not alter the main observation that the patient and tumor characteristics recorded could not explain why those who achieved OR did not obtain a longer TTF, but rather a tendency towards a shorter TTP. Differences in tumor grade is theoretically the most plausible explanation for any differences in TTP between the groups. We found no such difference between the groups with regards to the proliferation marker Ki67% although this could be due to the rather limited number of patients. We cannot know for sure whether the recorded Ki-67% estimates are representative for each of the patients. We know that there is significant intratumor heterogeneity [10] and that there are differences in Ki-67% between primaries and metastases [11]. We only have one to three Ki-67% estimates from each patient, and with disseminated disease, this estimate might not be representative for their disease. Some of the Ki-67% assessments were performed by less experienced pathologists and not all samples were reexamined by our institution’s pathologists specialized in neuroendocrine neoplasms. We do not believe, however, that occasional suboptimal evaluation of the proliferation index would systematically bias the assessment, but tend to both over- and underestimate the Ki67%, probably at the same extent.

The method used to assess radiological response in this study is both a strength and a limitation. The most widely used radiological response criteria for radiological response evaluation in treatment studies on neuroendocrine tumors are the Response Evaluation Criteria In Solid Tumors (RECIST 1.0 or 1.1) [12] the Southwest Oncology Group standard response criteria (SWOG) [13] and the WHO criteria [14]. These criteria were introduced to evaluate the effect of chemotherapy on tumor burden and are based on measuring the diameter of predefined target lesions as well as detection of any new lesions. In the RECIST-criteria, the most widely used assessment method, the diameters of the target lesions are added, and an increase from the start of treatment, or after initial therapy-induced tumor shrinkage, of 20% or more is defined as progressive disease. A reduction of 30% or more is defined as an objective response. Any change between 20% increase and 30% reduction is classified as stable disease. If new lesions emerge, or if preexisting non-target lesions grow, even if there is no change in the target lesions, the patient has progressive disease. RECIST is far from optimal for evaluating treatment response in slow-growing malignancies such as neuroendocrine tumors [15]. We have previously shown that assessing treatment response with RECIST gives an unrealistic positive impression of the treatment effect compared to assessing the treatment response with our “conventional method” [9]. The treatment response in the SD-group based on these criteria varies from a 19% increase to 29% decrease in added target lesion diameter. The heterogeneity in this group restricts our possibility to detect clinically interesting features as demonstrated in our study where the survival curves for those treated with PRRT overlaps when the RECIST criteria are used and diverges when evaluated with the “conventional method” (where any unequivocal change was regarded significant). The “conventional method” is, however, far from optimal. It lacks standardization and it is based on one or two radiologist’s overall impression of the tumor status. It is therefore not suitable in treatment trials or for reproducing results made by other investigators. Our results indicate, however, that radiological response evaluation systems that are more sensitive to response changes in neuroendocrine tumors are highly needed. With the high resolution of today’s radiological procedures one could argue that the thresholds used for classifying the different overall response groups in RECIST could be redefined. For example, 5% increase in the sum of diameters of target lesions instead of 20% could define progressive disease. Decreased tumor density as an effect of treatment secondary to tumor necrosis is not taken into account in the above mentioned response evaluation systems. Sometimes reduction in tumor viability, recognized as changes in contrast uptake, is the only initial sign of treatment effect. To meet these challenges in response evaluation irRECIST has been introduced [16]. The Choi criteria combine density and size with a lower size threshold than RECIST, and has been proposed for use in response evaluation for neuroendocrine tumors [17]. It has been shown to be more accurate compared to RECIST in a trial of sunitinib for gastroenteropancreatic neuroendocrine tumors [18]. Other response evaluation systems incorporating density and size as mRECIST, Chun and MASS [19–21] have so far not been validated for evaluation of treatment effect in neuroendocrine tumors. In the future we will probably also see that artificial intelligence with its ability to detect and interpret minor changes in size and density in the CT, MRI and PET examinations will be used in routine evaluations [22].

## Conclusion

For several tumor targeted therapies we found no benefit with regards to TTP for those who experienced OR compared to those who achieved SD.

## Data Availability

The datasets used and/or analysed during the current study are available from the corresponding author on reasonable request.

## Abbreviations

CT: Computed tomography
IQR: Interquartile range
5-FU: 5-fluorouracil
MRI: Magnetic resonance imaging
NET: Neuroendocrine tumors
OR: Objective response
PET: Positron emission tomography
PRRT: Peptide receptor radionuclide therapy
SD: Stable disease
SSA: Somatostatin analogue
Stz: Streptozocin
TTP: Time to progression
